# Female role models in analytical chemistry: then, now, and in the future

**DOI:** 10.1007/s00216-020-02763-w

**Published:** 2020-06-13

**Authors:** Antje J. Baeumner, Hua Cui, Maria Cruz Moreno Bondi, Sabine Szunerits

**Affiliations:** 1 Institute for Analytical Chemistry, Bio- and Chemosensors, University of Regensburg, Universitätsstraße 31, 93053 Regensburg, Germany; 2 Department of Chemistry, University of Science and Technology of China, 96 Jinzhai Road, Hefei, 230026 Anhui China; 3 Department of Analytical Chemistry, Faculty of Chemistry, Complutense University, 28040 Madrid, Spain; 4 Institut d’Electronique, de Microélectronique et de Nanotechnologie (IEMN), UMR CNRS 8520, Université de Lille, 59652 Villeneuve d’Ascq, France

“Who were your role models?” is a question we get asked seemingly at all relevant career stages of our scientific lives. Concerning women in chemistry, this question is often asked with more *precision* inquiring about our *female* role models in *chemistry*. This question is answered quickly—no need to think—because we have the fabulous, unparalleled in their historic successes, female chemists Marie and Irene Curie, and Rosalind Franklin, moving right along to Dorothy Hodgkin. All set! But, wait a moment, based on this superheroine foundation, we can dig deeper to answer this question with more *specificity* and *actuality*. Ada E. Yonath (2009) and Frances H. Arnold (2018), who were awarded Nobel Prizes in Chemistry for their outstanding scientific accomplishments, are more than obvious role models for so many of us. So let us keep going, because in addition we can identify with *accuracy* those important role models who did not inspire us from afar in history or afar from a Nobel worthy platform. Rather, they inspired us much more closely with their demonstrated ingenuity and creativity, persistence and success, and leadership accompanied with outstanding personalities. Here, choices become more personal, more focused on own fields of research, and are accompanied with the serendipity of having had a chance to meet these great women. We are talking about chemists of the caliber of Jacqueline K. Barton, Elizabeth (Lisa) A. H. Hall, Frances S. Ligler, Marja-Liisa Riekkola, Carol V. Robinson, and Dong Shaojun, to name a few of our truly outstanding female chemists who have served so many of us as role models scientifically‚ as well as personally‚ and whose excellence we aspire to emulate. Their excellence is underlined by having accomplished many “firsts” in their own universities and countries, by being recognized by their respective countries’ premier scientific academies, and by having contributed in major ways to their fields through seminal books and pioneering research.

While indeed the history of science is often read like a list of bearded old *men*, many incredible and *inspiring women* have changed science. It is hence with considerable optimism‚ and the general belief that these female pioneers are not only inspiring women but men alike, that this special ABC issue is devoted to presenting research performed by a selected list of early and advanced career female scientists. Undeniably, with the extremely rich and diverse scientific articles of the about 60 chosen female researchers contributing to classical analytical fields‚ such as mass spectrometry, chromatography, electrochemistry, and spectroscopy, and to more nanoscience-oriented approaches‚ such as molecular nanosensors, microchip analysis, and nanoparticles for sensing, this issue demonstrates that females are present in all areas of analytical science. It remains our duty to ensure the visibility of their excellent science, which is linked to their continuous efforts to maintain the highest standards of science. It seems like centuries away that we counted on only a handful of female chemists. Looking at this special issue, it is evident that‚ in 2020, an abundance of role models for current and future generations of analytical chemists is available.

The research included in this issue spans the globe and provides some insight into the breadth of analytical and bioanalytical chemistry at the forefront of the field. We thank all the researchers who contributed to the special collection, but especially to all the women who lead the research. With their creativity, dedication, and enthusiasm, they pave the way for the new generations of scientists working in this largely interdisciplinary field. A hundred years were enough to diversify gender-conventional ideas and reasoning. However, now is the time to envision a future with more women chemists leading the way and using that visibility to ensure that all of us, regardless of gender, work together to ensure the progress of our society at large.

